# Effect of Ni/Rh ratios on characteristics of Ni_x_Rh_y_ nanosponges towards high-performance hydrogen evolution reaction

**DOI:** 10.1016/j.dib.2019.103941

**Published:** 2019-04-20

**Authors:** Ngoc-Anh Nguyen, Ho-Suk Choi

**Affiliations:** Department of Chemical Engineering and Applied Chemistry, College of Engineering, Chungnam National University, 99 Daehak-ro, Yuseong-Gu, Daejeon, 34134, Republic of Korea

**Keywords:** Ni_x_Rh_y_ alloys, Nanosponges, Electrocatalysts, Hydrogen evolution reaction, Crystallinity

## Abstract

The data presented in this article are related to the research article entitled “NiRh Nanosponges with Highly Efficient Electrocatalytic Performance for Hydrogen Evolution Reaction (N.-A. Nguyen et al., 2019) [1]. This article reports a facile method to prepare various Ni_x_Rh_y_ nanosponges while using NaBH_4_ as a reducing agent without using any surfactant. The structural and chemical properties of the obtained Ni_x_Rh_y_ nanosponges are investigated.

**Table:** Specifications table

Subject area	*Physics, Chemistry*
More specific subject area	*Electrochemical catalysts for hydrogen evolution reaction*
Type of data	*Table, image (x-ray, microscopy, etc.), graph, figure*
How data was acquired	*The structure of the prepared samples is determined by Powder X-ray diffraction (XRD) measurement with a Cu target (Cu Kα1* = *1.541 Å), (Japan). The measurements are conducted from 20◦ to 80◦ with steps of 0.02◦. The morphology is observed by scanning electron microscopy (SEM, JSM-7000F, JEOL, Japan) and transmission electron microscopy (TEM, JEM-2100F, JEOL, Japan)). X-ray photoelectron spectroscopy (XPS) characterization is performed on a Sigma Probe Thermo Fisher VG Scientific spectrometer (MULTILAB* 2000*, Thermo Scientific, USA) equipped with a monochromatic Al Kα X-ray source.*
Data format	*Raw, filtered, analyzed*
Experimental factors	*Mole ratio of Ni:Rh*
Experimental features	*First,* 10 mL *of 0.1 M NaBH*_*4*_*is put in a three-neck flask. A mixture of* 2 mL *of 0.1 M NiCl*_*2*_*and RhCl*_*3*_*(mole ratio of Ni:Rh to be 3:1) is then quickly added to* 10 mL *of 0.1 M NaBH*_*4*_*solution above. After a 15 minute reaction, a black product is obtained through centrifuging and washing it three times in distilled water and three times in ethanol. This black product is then dried in an oven at a temperature of 80ºC for 12 hours to obtain the final product. To synthesize other samples such as Ni, Rh, Ni*_*1*_*Rh*_*1*_*, and Ni*_*1*_*Rh*_*3*_*, the same methods are applied except using different mole ratios of Ni and Rh: 1:0, 0:1, 1:1, and 1:3, respectively.*
Data source location	*Chungnam National University, Daejeon, South Korea*
Data accessibility	*Data is provided with this article*
Related research article	*Ngoc-Anh Nguyen, Van-Toan Nguyen, Sangho Shin, Ho-Suk Choi, “NiRh Nanosponges with Highly Efficient Electrocatalytic Performance for Hydrogen Evolution Reaction”, Journal of Alloys and Compounds, 789 (*2019*) 163–173.*[1]

**Table:** 

**Value of the data** • Ni_x_Rh_y_ nanosponge was synthesized by using NaBH_4_ as a reducing agent without using any surfactants. • HRTEM images are showed to see the effect of Ni/Rh ratios on the characteristics of prepared electrocatalysts. • Rh-rich NiRh alloy nanosponges possess the smallest average crystallite size. • The EDS and XPS spectra confirm that Ni and Rh atoms are alloying together in the prepared electrocatalysts. • HRTEM images exhibit that the morphology and the structure of Ni_1_Rh_3_ nanosponge are not changed after the stability test of the overwater splitting process.

## 1 Data

**Scheme 1:**
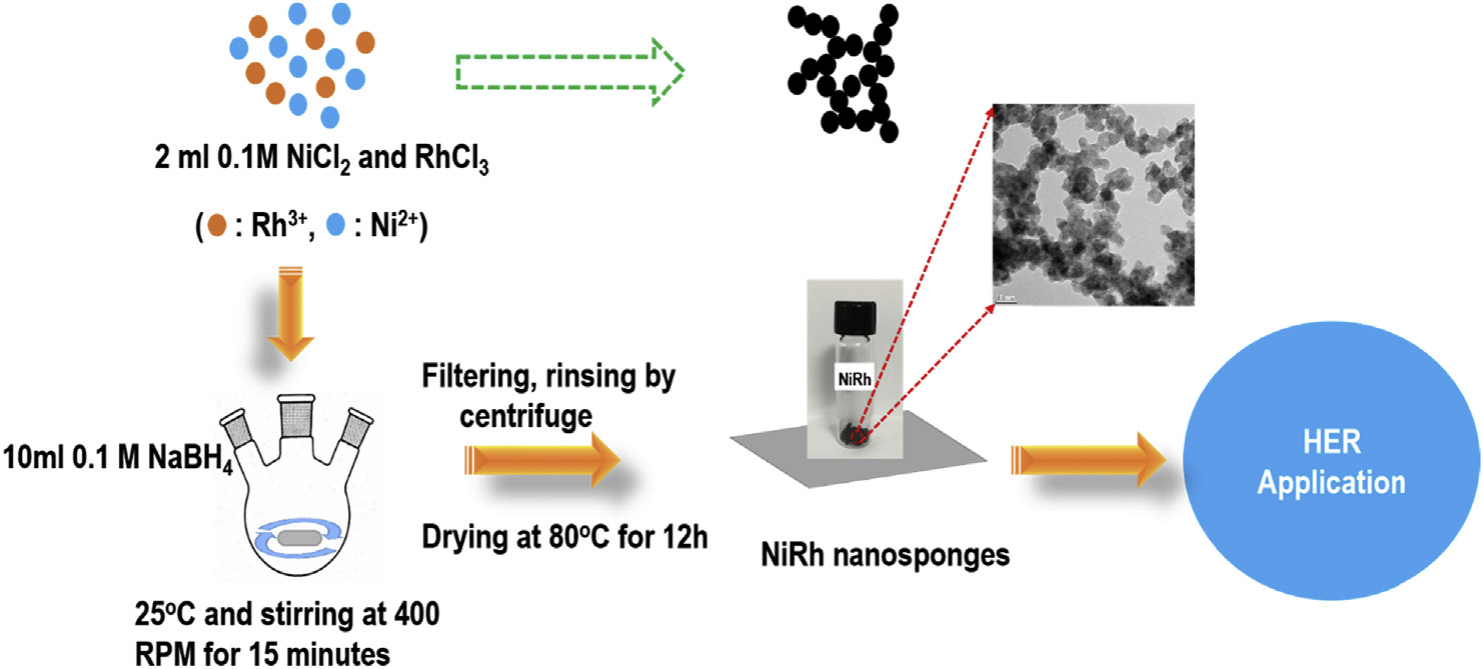
Schematic illustration of the synthesis process of NiRh nanosponges.

**Fig. 1:**
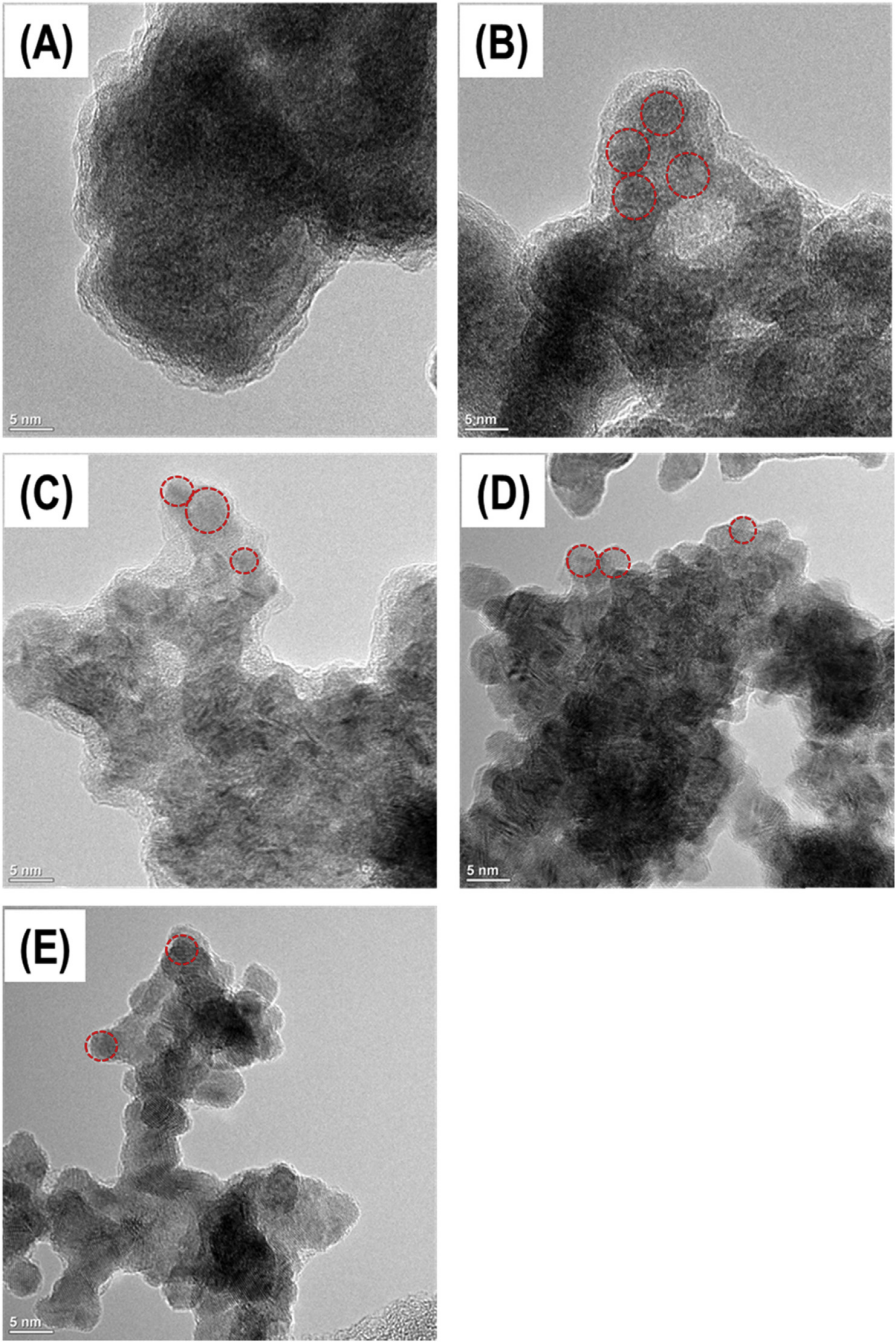
(A–E) HRTEM images show the morphology of Ni, Ni_3_Rh_1_, Ni_1_Rh_1_, Ni_1_Rh_3_, and Rh nanosponges, respectively.

**Fig. 2:**
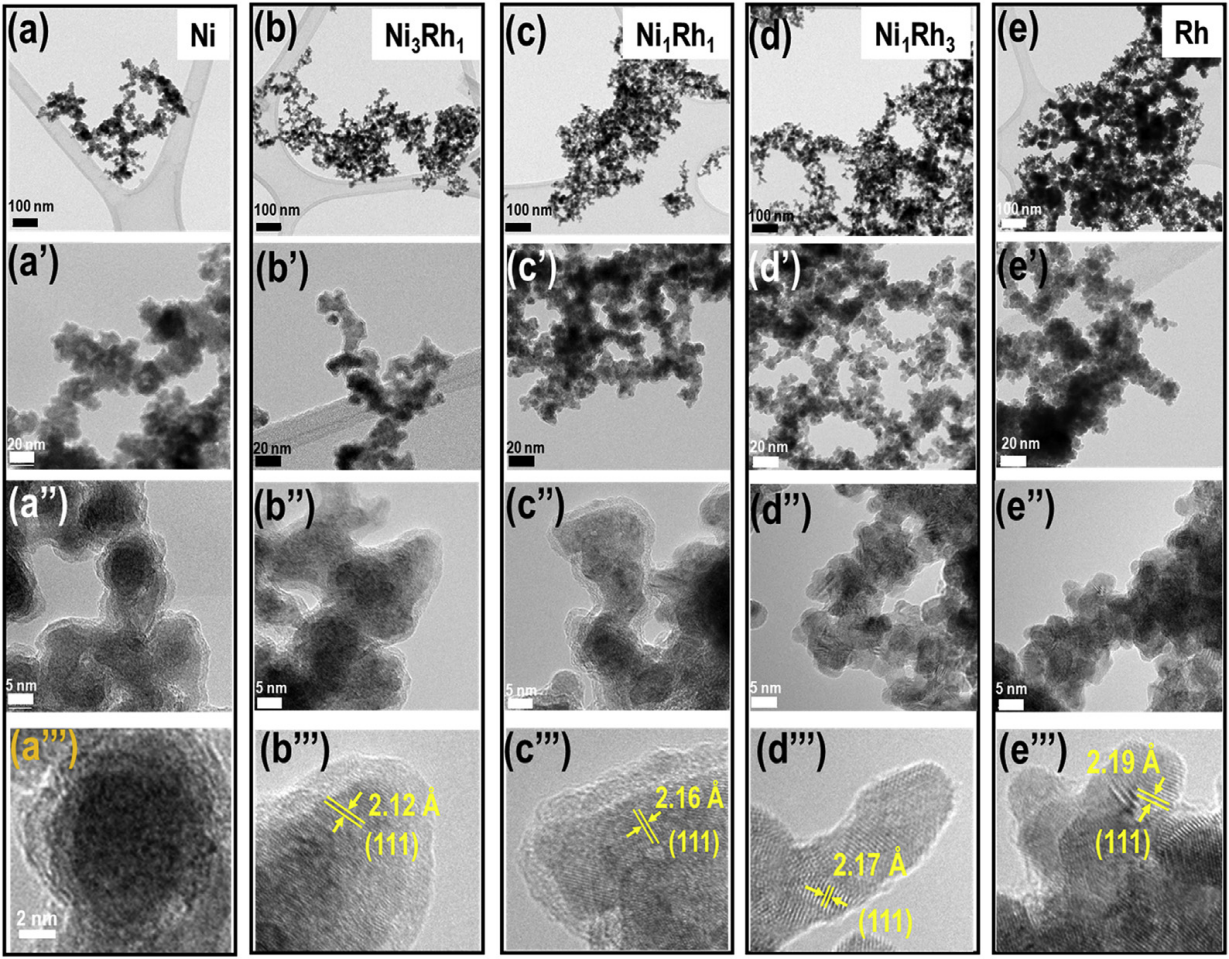
(a–e) TEM and HRTEM images exhibit the morphology and d-spacings of Ni, Ni_3_Rh_1_, Ni_1_Rh_1_, Ni_1_Rh_3_, and Rh nanosponges, respectively.

**Fig. 3:**
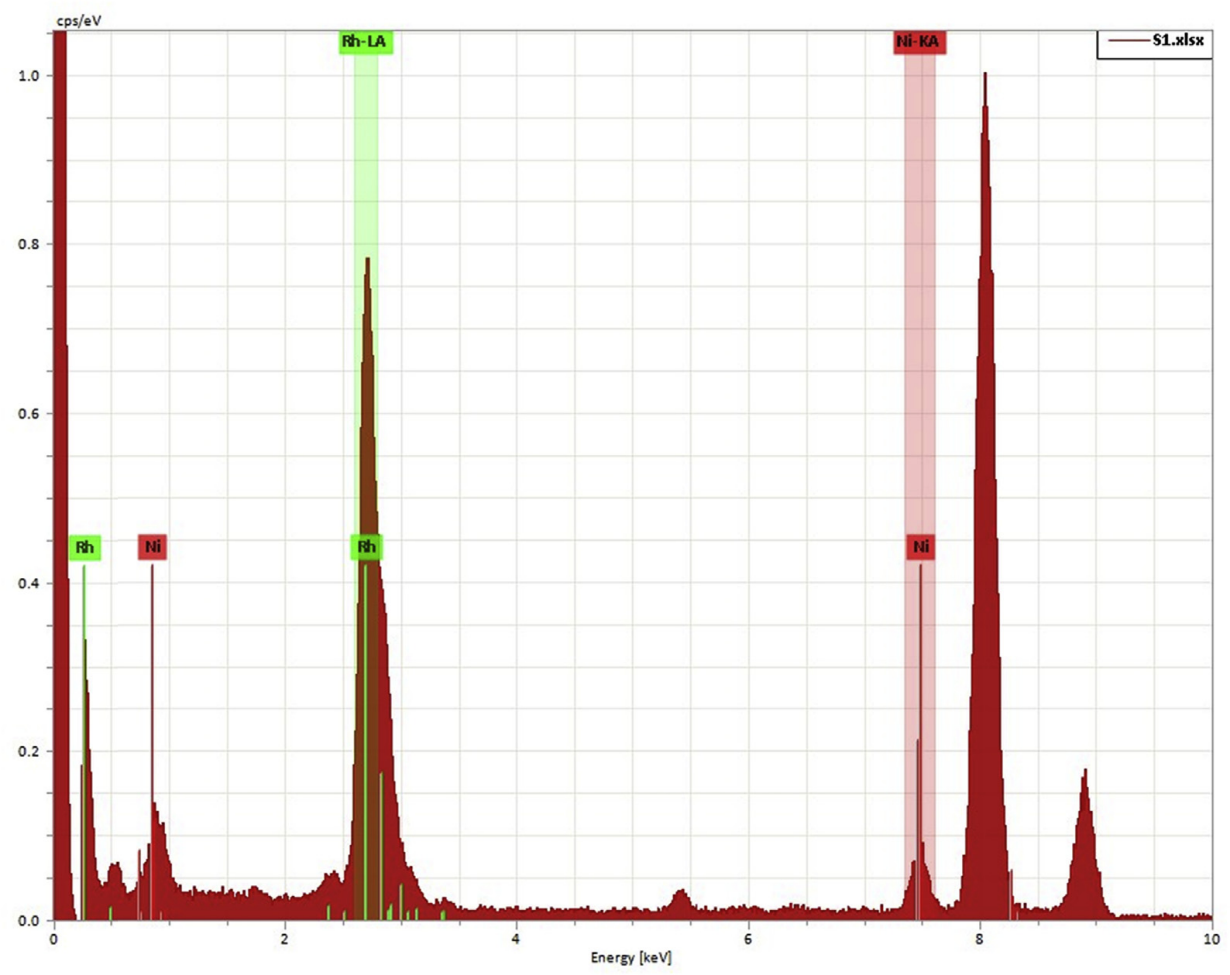
The EDS spectrum presents the appearance of Ni and Rh atoms of Ni_1_Rh_3_ nanosponge.

**Fig. 4:**
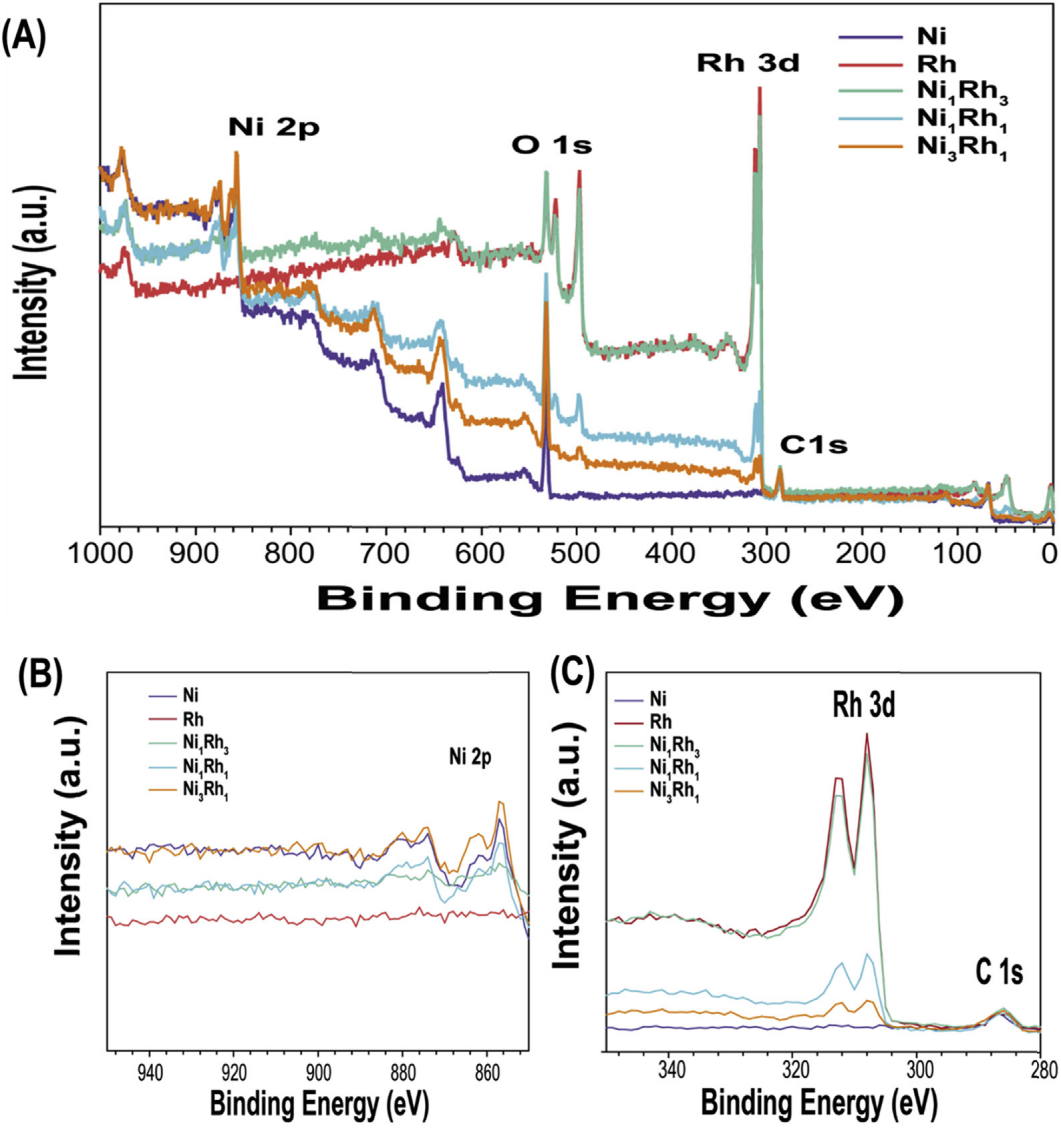
(A) Full XPS spectra, (B) XPS spectra of Ni 2p, (C) XPS spectra of Rh 3d of Ni, Ni_3_Rh_1_, Ni_1_Rh_1_, Ni_1_Rh_3_, and Rh, respectively.

**Fig. 5:**
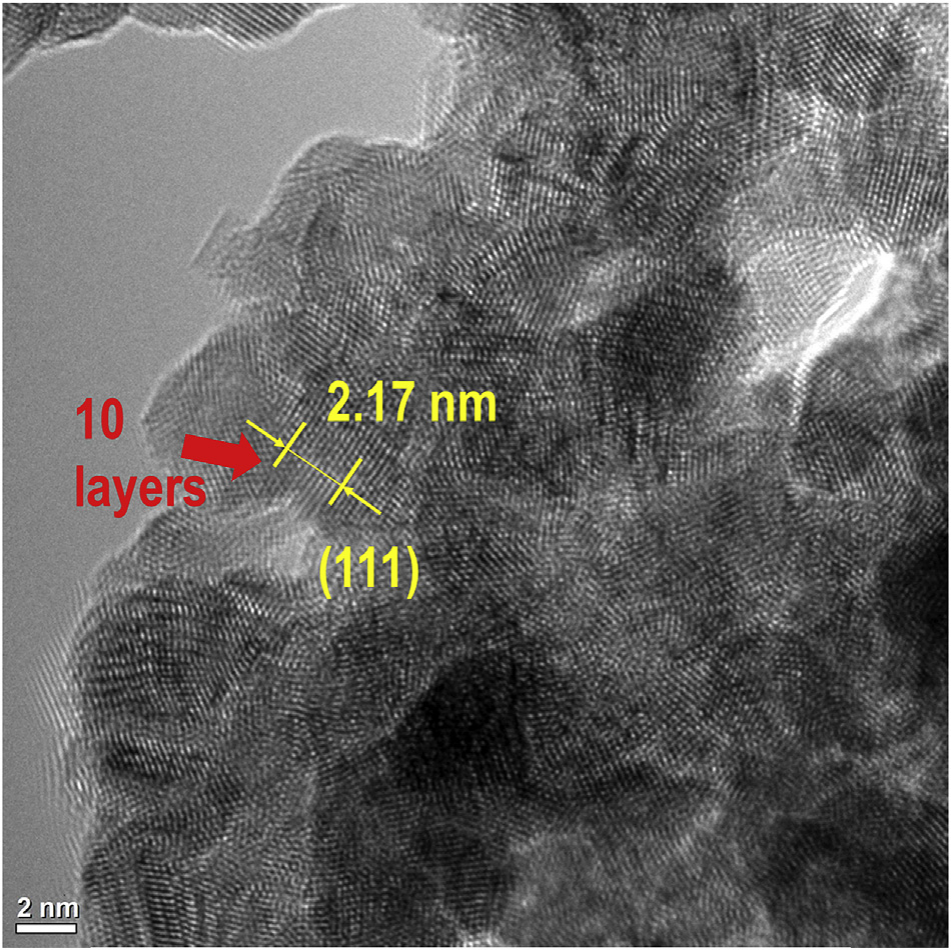
HRTEM image exhibits the stability in the morphology and structure of Ni_1_Rh_3_ nanosponge after the stability test in the overwater splitting process in 1.0 M KOH.

The data of this article provides information on the synthesis of various Ni_x_Rh_y_ nanosponges and their characteristics which effect on the performance of hydrogen evolution reaction (HER). Scheme 1 shows the synthesis of various Ni_x_Rh_y_ nanosponges. Fig. 1, Fig. 2, Fig. 3, Fig. 4 and Table 1 present the characterizations of various Ni_x_Rh_y_ nanosponges. Fig. 5 shows the TEM image of Ni_1_Rh_3_ nanosponge after the stability test from the pair of Ni_1_Rh_3_//RuO_2_ (both Ni_1_Rh_3_ and RuO_2_ were coated onto Ni foam (NF)) applied for the overall water splitting process in 1.0 M KOH electrolyte.

**Table 1:** The characterizations about 2θ (of a plane (111)), d-spacings (of a plane (111)), average crystallite sizes of various NiRh nanosponges.

Catalyst	2θ at plane (111) (^°^)	2θ of plane (200) (^°^)	FWHM (^°^)	D-spacing (111) (nm) (Calculated)	D-spacing (111) (nm) (Measured by TEM)	Average Crystalline size (nm) (Calculated)
Rh	41.23 ± 0.03	46.95 ± 0.05	2.80 ± 0.02	0.2187	0.2191 ± 0.0002	3.032
Ni_1_Rh_3_	41.41 ± 0.03	47.62 ± 0.03	2.95 ± 0.01	0.2179	0.2172 ± 0.0003	2.880
Ni_1_Rh_1_	41.46 ± 0.01	47.52 ± 0.05	2.65 ± 0.03	0.2176	0.2169 ± 0.0004	3.207
Ni_3_Rh_1_	42.35 ± 0.04	47.44 ± 0.04	1.81 ± 0.02	0.2132	0.2123 ± 0.0004	4.693

## 2 Experimental design, materials, and methods

### 2.1 Materials

Nickel (II) chloride hexahydrate (NiCl_2_.6H_2_O, 99,99% trace metal basic), Rhodium (III) chloride (RhCl_3_. xH_2_O, ≥99.9% trace metals basis), (NaBH_4_ powder, ≥98.0%), and Ruthenium (IV) oxide (RuO_2_, 99.9% trace metals basis) were purchased from Sigma Aldrich (USA). The Nafion D521 solution (5 wt%) was bought from Dupont (USA). Ni foam (≥99.5%) was supplied from Invisible Inc., Korea.

### 2.2 Methods

The synthesis process of Ni_x_Rh_y_ nanosponges (x:y = 1:0,3:1, 1:1, 1:3, and 0:1) is fully described in a scheme, which was given in Scheme 1. For preparing Ni_3_Rh_1_, the following steps have been conducted. First, 10 mL of 0.1 M NaBH_4_ is put in a three-neck flask. A mixture of 2 mL of 0.1 M NiCl_2_ and RhCl_3_ (mole ratio of Ni:Rh to be 3:1) is then quickly added to 10 mL of 0.1 M NaBH_4_ solution above. After a 15 minute reaction, a black product is obtained through centrifuging and washing it three times in distilled water and three times in ethanol. This black product is then dried in an oven at a temperature of 80 °C for 12 hours to obtain the final product. To synthesize other samples such as Ni, Rh, Ni_1_Rh_1_, and Ni_1_Rh_3_, the same methods are applied except using different mole ratios of Ni and Rh: 1:0, 0:1, 1:1, and 1:3, respectively.

### 2.3 Experimental design

To see the morphology of prepared Ni_x_Rh_y_ nanosponges, the HR-TEM images are exhibited for Ni, Ni_3_Rh_1_, Ni_1_Rh_1_, Ni_1_Rh_3_, and Rh nanosponges as seen in Fig. 1, which is consistent with the previous reports [2], [3], [4]. The TEM images imply that all Ni_x_Rh_y_ nanosponges are composed of fused nanoparticles with diameter from 10 to 20 nm. Fig. 2(a-a’)-(2e-e’) exhibit the sponge-like morphology for all Ni_x_Rh_y_ nanosponges. However, as seen in Fig. 2(a’‘-a’’‘)-(e’‘-e’’‘), it can be clearly observed that there are nanoparticles covered with a thin amorphous layer in the samples possessed the higher content of Ni (Ni, Ni_3_Rh_1_, and Ni_1_Rh_1_ samples). These thin amorphous layers may block the active sites, therefore, reducing the HER performance of the catalyst [1], [4]. In order to further understand the effect of Ni/Rh ratios on the characteristics of Ni_3_Rh_1_, Ni_1_Rh_1_, Ni_1_Rh_3_, and Rh samples (except for Ni sample that possessed an amorphous structure), we measured the d-spacings for all these samples by two different methods. At the first method, we obtained the d-spacings for Ni_3_Rh_1_, Ni_1_Rh_1_, Ni_1_Rh_3_, and Rh samples by using TEM measurement. At the second method, we calculated the d-spacings for Ni_3_Rh_1_, Ni_1_Rh_1_, Ni_1_Rh_3_, and Rh samples by using the Bragg's Law based on the XRD results. Specifically, the d-spacing is calculated by using Bragg's Law, as seen in Eq. (1):

(1) nλ = 2d sinθ

Where n is an integer; λ is the wavelength of incident light; d is a lattice spacing; θ is an angle of incidence.

The diffraction peak of (111) plane is used to estimate the nanoparticle size by the Debye-Scherrer Formula, as seen in Eq. (2) as follows:

(2) D = kλ/B cosθ

Where D is the average particle size (nm), k is a constant of 0.9, the wavelength (l) of an X-ray is equal to 0.154056 nm, B is the peak width at half height, and θ is the angle of (111) diffraction peak. The data in details are showed as seen in Table 1. According to these results, we realize that the sample of Ni_1_Rh_3_ nanosponge exhibits a higher degree of crystallinity and the lowest average crystalline size (2.880 nm) compared to the other samples. It can be used to explain why the highly HER performance in 0.5 M H_2_SO_4_ electrolyte for Ni_1_Rh_3_ sample compared to the other sample as seen in the article [1]. In which, the lowest value of an overpotential at a current density of 10 mA cm^−2^ is only 48 mV compared to Ni, Ni_3_Rh_1_, Ni_1_Rh_1_, and Rh samples (403, 75, 64, and 120 mV; respectively) [1]. To further understand the distribution of elements in Ni_1_Rh_3_ nanosponge, we tested the element mapping for this sample with the EDS analysis as seen in the article [1]. As a result, Ni and Rh can be seen in appearance and distribution uniformly in the sample. On the other hand, the XRD and XPS results are performed to confirm the Ni/Rh alloying together [1], [4], [5]. The full XPS spectra of all samples of Ni, Ni_3_Rh_1_, Ni_1_Rh_1_, Ni_1_Rh_3_, and Rh samples are given as seen in Fig. 4. Clearly, Ni metal does not appear in the Rh sample as well as Rh metal does not appear in the Ni sample (Fig. 4B and C). The shifted XPS peaks of Ni 2p and Rh 3d in the Ni_3_Rh_1_, Ni_1_Rh_1_, and Ni_1_Rh_3_ samples are explained due to the alloying between Ni and Rh in these samples. Finally, to see the change of structure of Ni_1_Rh_3_ sample, the TEM image is obtained for Ni_1_Rh_3_ nanosponge after the stability test of the pair of Ni_1_Rh_3_//RuO_2_ (both Ni_1_Rh_3_ cathode and RuO_2_ anode materials were coated onto Ni foam (NF)) applied for overall water splitting in 1.0 M KOH electrolyte. From this TEM image as seen in Fig. 5, we can conclude that the d-spacing of Ni_1_Rh_3_ nanosponge to be 2.17 nm, suggesting this catalyst is very stable after the water electrolysis process.
